# A Functional Analysis of the Spacer of V(D)J Recombination Signal Sequences

**DOI:** 10.1371/journal.pbio.0000001

**Published:** 2003-10-13

**Authors:** Alfred Ian Lee, Sebastian D Fugmann, Lindsay G Cowell, Leon M Ptaszek, Garnett Kelsoe, David G Schatz

**Affiliations:** **1**Howard Hughes Medical Institute, Section of Immunobiology, Yale University School of MedicineNew Haven, ConnecticutUnited States of America; **2**Department of Immunology, Duke University Medical CenterDurham, North CarolinaUnited States of America; **3**Ruttenberg Cancer Center, Mount Sinai School of Medicine of New York UniversityNew York, New YorkUnited States of America

## Abstract

During lymphocyte development, V(D)J recombination assembles antigen receptor genes from component V, D, and J gene segments. These gene segments are flanked by a recombination signal sequence (RSS), which serves as the binding site for the recombination machinery. The murine Jβ2.6 gene segment is a recombinationally inactive pseudogene, but examination of its RSS reveals no obvious reason for its failure to recombine. Mutagenesis of the Jβ2.6 RSS demonstrates that the sequences of the heptamer, nonamer, and spacer are all important. Strikingly, changes solely in the spacer sequence can result in dramatic differences in the level of recombination. The subsequent analysis of a library of more than 4,000 spacer variants revealed that spacer residues of particular functional importance are correlated with their degree of conservation. Biochemical assays indicate distinct cooperation between the spacer and heptamer/nonamer along each step of the reaction pathway. The results suggest that the spacer serves not only to ensure the appropriate distance between the heptamer and nonamer but also regulates RSS activity by providing additional RAG:RSS interaction surfaces. We conclude that while RSSs are defined by a “digital” requirement for absolutely conserved nucleotides, the quality of RSS function is determined in an “analog” manner by numerous complex interactions between the RAG proteins and the less-well conserved nucleotides in the heptamer, the nonamer, and, importantly, the spacer. Those modulatory effects are accurately predicted by a new computational algorithm for “RSS information content.” The interplay between such binary and multiplicative modes of interactions provides a general model for analyzing protein–DNA interactions in various biological systems.

## Abstract 2

Spacers not only ensure that the distance between the nonamer and heptamer is correct but they also regulate recombination activity by providing protein-binding sites along the DNA sequences that affect recombination

## Introduction

During B- and T-lymphocyte development, the immunoglobulin (Ig) and T-cell receptor (TCR) genes are assembled from discrete V, D, and J gene elements via a process of genomic rearrangements known as V(D)J recombination (Fugmann et al. 2000a; Hesslein and Schatz 2001). V(D)J recombination occurs in two steps: a cleavage phase, in which DNA double-strand breaks are created, followed by a joining phase (Fugmann et al. 2000a). During cleavage, the lymphoid-specific recombinase proteins, RAG1 and RAG2, presumably together with the accessory DNA-binding factor HMG-1/2, bind recombination signal sequences (RSSs) located adjacent to each rearranging gene element. A complex consisting of RAG and HMG proteins bound to a single RSS is then thought to capture a second RSS (Jones and Gellert 2002; Mundy et al. 2002); within this synaptic complex, the RAG proteins introduce double-strand breaks at the junctions between each RSS and its associated gene element (Hiom and Gellert 1998). In the joining phase, ubiquitous DNA repair factors involved in nonhomologous end joining, in the presence of the RAG proteins, ligate the cleaved ends, generating two types of recombinant junctions: precise signal joints (SJs) and imprecise coding joints (CJs) (Bassing et al. 2002).

RSSs are an essential part of V(D)J recombination, as their presence is both necessary and sufficient to direct RAG-mediated recombination on artificial substrates. Sequence alignments of RSSs suggested that each signal can be dissected into three components: a conserved heptamer (consensus: 5′-CACAGTG) and a conserved nonamer (consensus: 5′-ACAAAAACC), separated by a poorly conserved spacer of either 12 ± 1 or 23 ± 1 bp (Tonegawa 1983; Akira et al. 1987; Ramsden et al. 1994). The heptamer is the site of DNA cleavage (Roth et al. 1992), while the nonamer provides a major binding surface for RAG1 (Difilippantonio et al. 1996; Spanopoulou et al. 1996; Nagawa et al. 1998; Swanson and Desiderio 1998). Spacer length restricts recombination according to the “12/23 rule”; efficient recombination occurs between two gene elements only when one element is flanked by an RSS with a 12 bp spacer (12-RSS) and the other by an RSS with a 23 bp spacer (23-RSS) (Tonegawa 1983).

Despite the enormous specificity that RSSs confer on the recombination process, the recombination signals themselves demonstrate a remarkable degree of sequence heterogeneity. Only the first three nucleotides of the heptamer and the fifth and sixth positions of the nonamer show almost perfect conservation (Ramsden et al. 1994) and are therefore thought to be the major determinants of RSS specificity and function. Mutations in any of these five “critical” nucleotides, alone or in combination, essentially abolish recombination (Tonegawa 1983; Akira et al. 1987; Hesse et al. 1989). The roles of the remaining “noncritical” heptamer and nonamer nucleotides are less understood. Some studies observed that mutations in these lesser-conserved residues have comparatively milder phenotypes unless present in combination (Tonegawa 1983; Hesse et al. 1989). Others, however, reported that nonconsensus deviations of noncritical residues lead to vastly different recombination efficiencies, resulting in significant differences in gene element usage in the unselected antigen receptor repertoire (Ramsden and Wu 1991; Suzuki and Shiku 1992; Connor et al. 1995; Larijani et al. 1999).

Our current knowledge about the functional role of the spacer is that its length is crucial in directing V(D)J recombination (Tonegawa 1983; Hesse et al. 1989). Comprehensive sequence alignments show that the spacer possesses some degree of sequence conservation, albeit at a level much lower than that of the heptamer or nonamer (Ramsden et al. 1994). This suggests that there is little or no selective pressure for spacers to adopt a given sequence. Studies examining the effects of different spacer sequences on recombination activity have yielded seemingly conflicting results. An early report found up to a 15-fold effect of different spacer sequences (Akira et al. 1987), while follow-up studies observed either no effect (Wei and Lieber 1993; Akamatsu and Oettinger 1998) or up to 6-fold effects (Fanning et al. 1996; Nadel et al. 1998; Larijani et al. 1999). This suggests that spacer sequence may affect recombination activity, but a comprehensive picture of the rules that govern how it does so is lacking.

One limitation inherent in many prior RSS studies is that they have often been performed in the context of RSSs with a preponderance of consensus nucleotides. While such analyses have been useful in characterizing the most conserved or critical determinants of RSS function, the contributions of other nucleotides are potentially masked in RSSs with high consensus nucleotide representation. That most endogenous RSSs do not contain consensus heptamer and/or nonamer motifs further suggests the need for a careful study of individual RSS nucleotides in the context of physiologically relevant RSSs.

**Figure 1:**
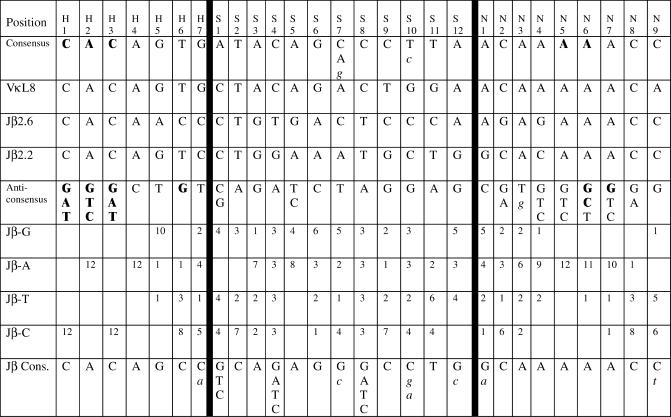
Recombination Signal Sequences Heptamer, spacer, and nonamer elements of 12-RSSs referred to in this study are shown. “Cons.” and “Anti-Cons.” denote the consensus and anticonsensus 12-RSSs, respectively. VκL8, Jβ2.6, and Jβ2.2 are murine 12-RSSs. “Jβ Cons.” denotes the consensus RSS compiled for all functional 12-RSSs in the murine Jβ1 and Jβ2 clusters. Where more than one nucleotide is listed at any given position, this indicates a shared preponderance of those nucleotides. For consensus RSSs, nucleotides in bold indicate almost absolute conservation; for the anticonsensus RSS, bold nucleotides are almost completely absent. Nucleotides in lowercase italics appear at slightly reduced frequencies compared to the other nucleotides listed. “Jβ-G/-A/-T/-C” and the corresponding numbers indicate the number of functional RSSs in the murine Jβ1/Jβ2 clusters at which the respective nucleotide appears at the designated position. At the top of the figure, the position of each nucleotide is labeled with respect to the first position of the respective element.

We have performed an extensive analysis of the functional properties of RSS elements in the context of endogenous recombination signals. To explore the nature of the complex relationships that might exist among different elements and positions in the RSS, we started with the nonfunctional RSS associated with the murine Jβ2.6 pseudogene element of the TCRβ locus (Jβ2.6 RSS). While most such pseudogene elements are flanked by RSSs with crippling mutations (Akira et al. 1987), Jβ2.6 is unique in that the sequence of its flanking RSS suggests no obvious explanation for its complete lack of activity (Figure 1). All of the critical residues are conserved, and each nonconsensus nucleotide in the heptamer and nonamer is represented in at least one other functional RSS in the TCRβ locus (Figure 1). A systematic analysis of Jβ2.6/consensus hybrid RSSs revealed that the nonamer, by itself, is the biggest determinant of Jβ2.6 RSS activity and that the lack of Jβ2.6 RSS function is due to the concerted action of nonconsensus nucleotides throughout the entire RSS, including the spacer. Surprisingly, we found that in combination with other consensus elements, an artificial consensus spacer can markedly boost recombination activity, while an anticonsensus spacer strongly impairs activity. Furthermore, in a genetic screen for functional spacer sequences, we observe a selective pressure for substrates with an increased representation of consensus nucleotides. Our results provide strong support for the model that RSS activity is a summation of numerous complex interactions between the RAG proteins and the RSS, involving not only the heptamer and nonamer but also most (if not all) basepairs of the spacer.

## Results

### In Vivo Assay for Recombination

**Figure 2:**
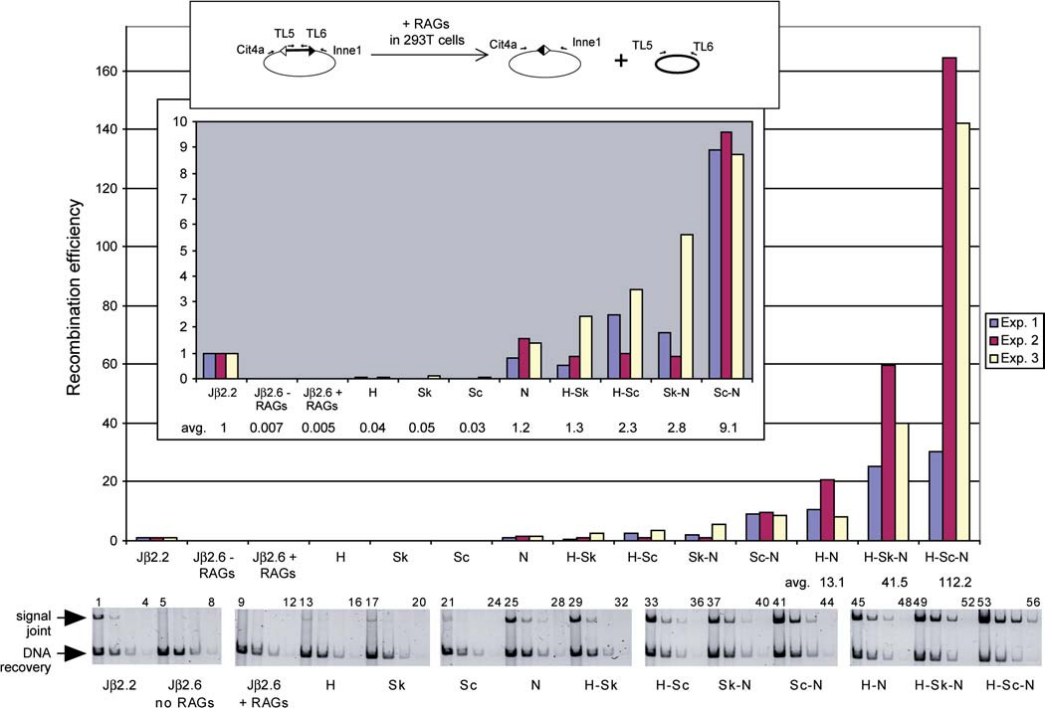
Recombination Activities on Hybrid Jβ2.6/Consensus RSSs A diagram of the recombination assay (SJ formation) is shown (top). Activities were measured on substrates containing the indicated hybrid 12-RSS and a standard 23-RSS. H, Sk, Sc, or N denotes the consensus heptamer, VκL8 spacer, consensus spacer, or consensus nonamer, respectively; each 12-RSS bears the indicated combination of consensus/VκL8 elements, with the remaining elements belonging to Jβ2.6 RSS. To determine relative recombination efficiencies, the amount of SJs was first corrected for DNA recovery, then normalized to the values obtained for the substrate containing the Jβ2.2 RSS. Relative recombination efficiencies for each of three experiments are shown as bar graphs; the average value is shown below each sample. The gels shown here correspond to Experiment 3 and represent products of PCRs on 10-fold dilutions of recovered plasmid DNA.

We generated a series of recombination substrates to measure the ability of various hybrid Jβ2.6/consensus 12-RSSs to rearrange to a “standard” 23-RSS (consisting of consensus heptamer and nonamer elements flanking a spacer from the functional Ig Jκ1 RSS). This standard 23-RSS was used instead of the natural Jβ2.6 RSS partner (the 23-RSS flanking Dβ2), since the substrates containing the Dβ2 23-RSS showed much lower levels of recombination in our hands (data not shown). The 12-RSS coding flank was the same for all constructs, namely that of Jβ2.6. For our study, a polymerase chain reaction (PCR)-based assay (Figure 2, top) was employed, which allowed us to visualize recombination efficiencies across a >1,000-fold range. The recombination substrates were transfected into the human embryonic kidney cell line 293T along with constructs expressing full-length RAG1 and RAG2 proteins, and recombination frequencies were measured by PCR using primers that amplify SJs. To confirm that the amplified products in our PCR assay were bona fide SJs, we demonstrated that they could be cleaved efficiently with *Apa*LI restriction endonuclease, which cuts precise RSS–RSS junctions (data not shown). The amount of recombination substrate recovered from each transfection was measured by PCR and used to normalize the recombination activity. Although we assayed primarily for SJ formation, analyses of CJ formation yielded parallel results (data not shown). As a reference, we used a substrate containing the 12-RSS from the TCR Jβ2.2 gene element (see Figure 1), which recombines at low but detectable levels, as measured both in our system and during T-lymphocyte development (Figure 2, lanes 1–4) (Livàk et al. 2000).

### Consensus Heptamer, Spacer, and Nonamer Replacements

Recombination of Jβ2.6 RSS is below the level of detection of our assay (Figure 2). Substitution of a consensus heptamer (H) into the Jβ2.6 RSS elevates the recombination frequency to levels just above background (Figure 2, lanes 13–16). Similarly, substitution of a spacer from a standard, functional 12-RSS (recombination signal sequence spacer [Sk], from Ig VκL8; see Figure 1) or of an artificial consensus spacer (Sc) only marginally restores recombination (Figure 2, lanes 17–24). By contrast, substitution of a consensus nonamer (N) boosts recombination activity to the level of Jβ2.2 RSS (Figure 2; compare lanes 1–4 to 25–28), approximately 20-fold higher than substitution of H, Sk, or Sc alone and at least two orders of magnitude above Jβ2.6 RSS. Therefore, the nonamer, by itself, is the biggest single determinant of Jβ2.6 RSS activity. The combination of a consensus heptamer and nonamer (H–N) further increases activity approximately 10-fold above N alone (Figure 2, lanes 45–48). Hence, the cumulative effects of nonconsensus mutations in the heptamer and nonamer elements of Jβ2.6 RSS are quite large.

In combination with a consensus heptamer and/or a consensus nonamer, the presence of either the VκL8 or the consensus spacer markedly enhances recombination activities above those observed with the Jβ2.6 RSS spacer (Figure 2, lanes 29–44). Although there is some fluctuation between experiments, in each replicate the greatest enhancement by the Sk or Sc spacer is seen in combination with a consensus heptamer: on average, H–Sk and H–Sc are 30- to 50-fold higher than H alone. By comparison, Sk–N and Sc–N are 3- to 8-fold higher than N, while H–Sk–N and H–Sc–N are 3- to 9-fold higher than H–N. Thus, a functional spacer can, in most cases, “rescue” the effects of a nonconsensus nonamer more fully than the effects of a nonconsensus heptamer, suggesting that the spacer has greater functional overlap with the nonamer than with the heptamer.

### Single-Nucleotide Consensus Replacements

The heptamer and nonamer of Jβ2.6 RSS differ from the consensus in only five positions (see Figure 1): the last three nucleotides of the heptamer and the second and fourth nucleotides of the nonamer. To determine which of these nucleotides make the greatest contributions to Jβ2.6 RSS activity, we introduced the respective consensus nucleotides individually at each of these positions. Since substitution of a consensus heptamer alone yields very low recombination levels (Figure 2), we assayed single-nucleotide heptamer replacements (H[5], H[6], and H[7]) in combination with a consensus spacer. We also assayed substrates containing H(5) combined with a consensus nonamer or with both consensus spacer and nonamer elements. All single-nucleotide heptamer replacements result in significant partial restoration of activity, to levels at least 50% of those obtained with the full consensus heptamer (data not shown). This suggests that the low activity of the Jβ2.6 RSS heptamer is due to contributions of all three nonconsensus nucleotides.

**Figure 3:**
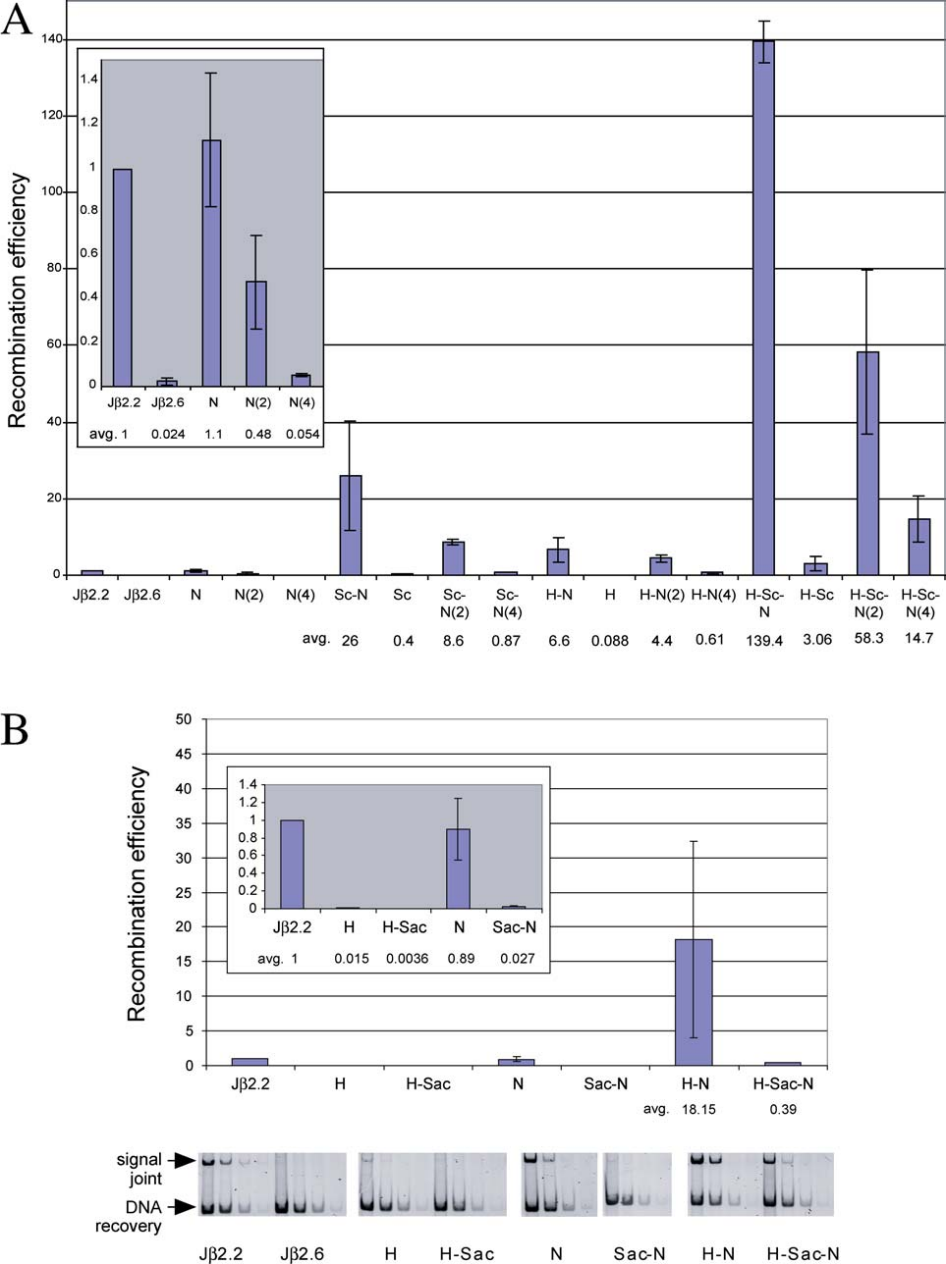
In Vivo Recombination Activities on Hybrid 12-RSSs with Nonamer Point Mutations or with the Anticonsensus Spacer The plots, error bars, and values listed below each sample represent the averages of three experiments. Note that all recombination efficiencies presented in this figure were obtained from transfections/PCRs that were completely independent from those shown in Figure 2. Abbreviations are identical to those used in Figure 2. (A) N(2) or N(4) denotes point substitution of the consensus nucleotide at the second or fourth position of the nonamer, respectively. (B) Sac indicates substrates that contain an anticonsensus 12-RSS spacer.

Substitution of a consensus nucleotide at either the second or fourth position of the nonamer (N[2] or N[4], respectively), alone or in combination with a consensus heptamer and/or spacer, partially reproduces the effects of the full consensus nonamer (Figure 3A). Interestingly, in each set of constructs, N(2) confers a greater restoration of activity than N(4): on average, constructs containing N(2) recombine at 50% the level of N, while constructs containing N(4) recombine at roughly 10% of N. This suggests that the recombination process has a greater preference for preserving a consensus C at the second position of the nonamer than a consensus A at the fourth position.

### Anticonsensus Spacer Replacements

In the presence of a consensus heptamer and/or nonamer, a consensus spacer markedly enhances recombination levels over the Jβ2.6 RSS spacer. We therefore wondered whether the presence of an artificial anticonsensus spacer (Sac) (see Figure 1), containing the least-conserved nucleotide at each position (Ramsden et al. 1994), would impair recombination. In all cases, Sac reduced recombination levels 10- to 20-fold compared to the already inefficient Jβ2.6 RSS spacer (Figure 3B; compare N to Sac–N, and H–N to H–Sac–N). In our experimental system, the consensus and anticonsensus spacer sequences are therefore capable of specifying a surprisingly large range of recombination efficiencies of up to two orders of magnitude.

### Coupled Cleavage In Vitro

**Figure 4:**
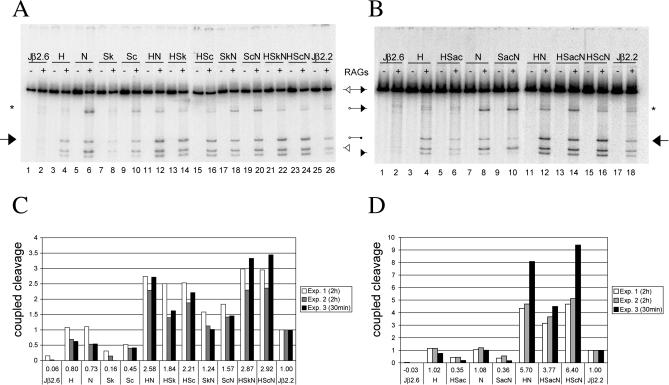
In Vitro Cleavage Reaction (A and B) Coupled cleavage was performed using body-labeled DNA substrates containing a standard 23-RSS (filled triangle) and different 12-RSSs (open triangle) as indicated above the lanes. Reaction products were separated on 4% polyacrylamide gels. The identity of the bands is indicated by symbols located between the gels; an arrow indicates the double cleavage product, while an asterisk marks single-site cleavage products. The gels shown here correspond to Experiment 2. (C and D) The intensity of the bands from three individual experiments (see legend) was quantified and the average cleavage efficiency calculated for each individual substrate (indicated below the chart). The efficiencies are displayed as relative to those obtained for Jβ2.2, which were arbitrarily set to 1.

Two important questions arise from the results of these in vivo assays. First, do the differences in the RSS nucleotide sequences affect the cleavage or the joining phase of the reaction? Second, are the RAG proteins by themselves the only proteins that mediate the discrimination between various RSSs? To address these questions, we performed standard 12–23 coupled cleavage reactions using purified, truncated (core) RAG proteins (Figure 4A). The linear substrates for these reactions were amplified by PCR from the plasmids used in the transient recombination assay. The amount of coupled cleavage products from three independent sets of reactions was quantified (Figure 4C). While the consensus RSS (H–Sc–N) promotes efficient cleavage of up to 23% of the input substrate, the Jβ2.6 RSS is cleaved at extremely low levels, at or below the limit of detection (Figure 4A, lane 2). As expected from the in vivo experiments, Jβ2.2 is sufficient for low but clearly detectable cleavage (Figure 4A, lane 26). In agreement with the SJ formation data, the consensus nonamer substitution (N) boosts the level of cleavage significantly (Figure 4A, lane 6), while the introduction of Sk or Sc has less effect (Figure 4A, lanes 8 and 10). In contrast to our findings on SJ formation, the substrate containing a consensus heptamer (H) is as efficiently cleaved as that containing N (Figure 4A; compare lanes 4 and 6). Interestingly, all substrates containing a consensus nonamer (and to a lesser extent those harboring a consensus spacer) show a high level of single-site cleavage at the 12-RSS (Figure 4A, lanes 6, 10, 12, 18, and 20); such products, which are only rarely generated on extrachromosomal substrates in vivo (Steen et al. 1997), could account for a reduced level of coupled cleavage compared to the recombination efficiencies obtained for the respective constructs in our SJ assays. The underlying mechanism of this phenomenon is the topic of ongoing studies.

Interestingly, a favorable spacer sequence (Sk or Sc), when paired with H or N, boosts cleavage over the Jβ2.6 RSS spacer (Figure 4A, lanes 12, 14, 16, and 18). The levels of cleavage for H–Sk or H–Sc are reproducibly higher than those for Sk–N or Sc–N; although the effect is less striking than for SJ formation, the limits of detection in the coupled cleavage assay dictate that this assay spans a much narrower range of activities than the SJ formation assay. To further address the role of spacer sequences in our coupled cleavage system, we performed another set of experiments using the substrates containing the anticonsensus spacer (Sac) (Figure 4B and 4D). In conjunction with either consensus heptamer (H–Sac) or consensus nonamer (Sac–N), the anticonsensus spacer reduces cleavage 5- to 10-fold compared to the consensus spacer (H–Sc or Sc–N) (Figure 4C and 4D) and 3-fold compared to the Jβ2.6 RSS spacer (H or N) (Figure 4B; compare lanes 4 and 8 to lanes 6 and 10, respectively). This suggests that the Jβ2.6 RSS spacer, although “poor” compared to Sk or Sc, is still more proficient for cleavage than Sac.

### RSS Binding

**Figure 5:**
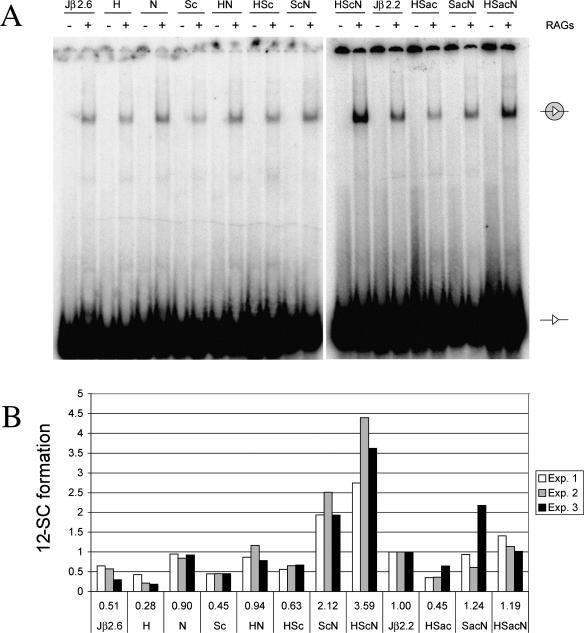
In Vitro Binding (A) Binding assays were performed using the 5′-end-labeled 12-RSS substrates indicated above the lanes. Each reaction contained identical amounts of DNA substrate. Owing to differences in the end-labeling efficiencies, the quantitation (shown in [B]) is required to make quantitative comparisons. The gels shown here correspond to Experiment 3. (B) The relative amount of substrate in the shifted complex was determined. The binding efficiencies from three independent experiments were calculated relative to the binding seen for Jβ2.2 oligonucleotides (which were arbitrarily set to 1). The average value is displayed below the chart.

It is likely that differences in the nucleotide sequences of the RSS lead to variations in the stability of RAG–RSS complexes (Hiom and Gellert 1997; Akamatsu and Oettinger 1998; Swanson and Desiderio 1998). This idea provides one obvious explanation for the observed differences in SJ formation and cleavage efficiency among the various analyzed 12-RSSs. To address this possibility, we analyzed binding of the RAG proteins to individual isolated 12-RSSs, since the 23-RSS remained identical in all experiments described above. Binding was assessed in standard gel-shift assays using oligonucleotide substrates containing the respective 12-RSSs (Figure 5A). All binding assays were performed three times; the quantitation of binding for each RSS relative to Jβ2.2 is displayed in Figure 5B. (Note that the amount of shifted complex has been normalized for the amount of free probe, which contributes to the fact that, between some samples, visual assessment of relative binding activities are less striking than quantitative measurements.) As expected, the consensus 12-RSS (H–Sc–N) shows the highest binding efficiency, while binding to the endogenous Jβ2.6 RSS is weak, about 2-fold reduced compared to our standard, the functional Jβ2.2 12-RSS. Given that, as with the coupled cleavage assay, the range of activities in the binding assay is much narrower than in the SJ formation assay, these results correlate well with those obtained in the other assays. Substitution of the individual consensus elements H, Sc, and N, however, led to surprising results. While the consensus nonamer (N) sequence, as expected, increases the level of binding (up to that of Jβ2.2), the consensus spacer (Sc) alone has no effect on binding at all, and the consensus heptamer (H) consistently reduces the level of binding. The consensus spacer boosts binding only in the context of a consensus nonamer (the ratios of Sc–N:N and H–Sc–N:H–N are greater than H–Sc:H), and the consensus heptamer contributes significantly to RAG–RSS interactions in this assay only when both spacer and nonamer are consensus sequences (H–Sc–N:Sc–N > H–N:N or H:Jβ2.6 RSS). This indicates that the nonamer is the predominant element determining the stability of the initial RAG–HMG–RSS complex while the heptamer makes additional important contributions to cleavage and recombination not reflected in this binding assay.

In the context of a consensus nonamer, the consensus spacer reproducibly enhances binding more than a consensus heptamer (Sc–N > H–N). In contrast, the anticonsensus spacer (H–Sac–N) reduces binding about 3-fold compared to H–Sc–N (Figure 5A and 5B). The effects of Sc–N compared to Sac–N are also clearly visible. Interestingly, the levels of binding in the presence of Sac are very similar to those obtained for the respective RSSs containing the original Jβ2.6 RSS spacer, in contrast to the comparative effects of the two spacers on cleavage (see Figure 4).

Taken together, the results of our binding studies underline clearly that the reduced ability of the Jβ2.6 RSS to participate in the initial interaction with the RAG complex, and hence the subsequent steps of V(D)J recombination, is caused not solely by the Jβ2.6 RSS nonamer but also by the “inefficient” spacer sequence. This indicates that the spacer helps the nonamer to efficiently lock the RAG proteins onto the RSS. The heptamer can contribute to this only when interactions with the other two elements are favorable.

### Genetic Screen for Functional Spacer Sequences

Although the RSS spacer is poorly conserved and no naturally occurring RSS has yet been identified that bears the published consensus spacer sequence, our results show that the presence of the most- or least-conserved nucleotides at all positions of the spacer dramatically alters recombination activities of RSSs that contain a consensus heptamer and/or nonamer. This suggests that a functional preference exists for certain spacer sequences over others. We therefore established a genetic screen for functional spacer sequences in which each position of the spacer was randomized to contain either a consensus or an anticonsensus nucleotide (Sc/Sac). Because the greatest effect of the consensus spacer in our experiments is seen in combination with a consensus heptamer (H–Sc), the randomized spacer was analyzed in the context of 12-RSSs containing a consensus heptamer and the Jβ2.6 RSS nonamer (H–Sc/Sac). The H–Sc/Sac library contained roughly 80,000 clones, sufficient to represent each of the 4,096 possible spacer sequences multiple times (data not shown).

**Figure 6:**
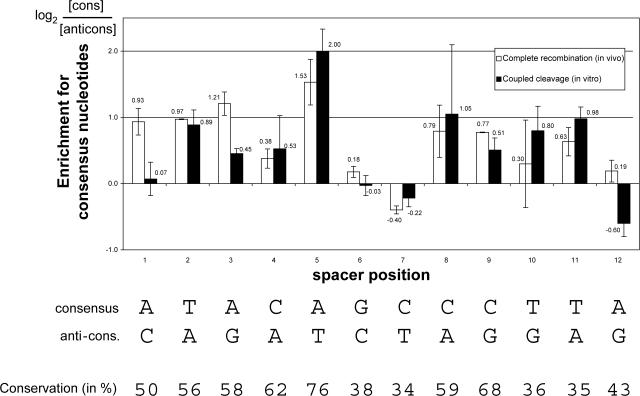
Genetic Screen for Preferred Spacer Sequences A plasmid library containing 12-RSSs with a consensus heptamer and either consensus or anticonsensus nucleotides at each position of the spacer was screened for spacers with higher activity using either in vivo recombination or in vitro coupled cleavage assays (see text for details). **** The number of library clones screened was >10^5^.**** In total, 240 sequences from two independent in vivo experiments and 205 sequences from two in vitro screens were analyzed. The relative enrichment for a consensus over an anticonsensus nucleotide at each position was calculated (taking the bias in the starting library into account). The average from two experiments is displayed in the bar graph and the values are displayed above or below the bars. The log_2_ of the ratio of the frequency of consensus and anticonsensus nucleotides at each position is displayed; hence, a value of one indicates that the respective nucleotide occurs two times more frequently in the selected population than in the starting library. In addition, the degree of conservation of each nucleotide is indicated (Ramsden et al. 1994).

We transfected the H–Sc/Sac library into 293T cells together with vectors expressing full-length *RAG1* and *RAG2*, and we cloned and sequenced PCR-amplified SJs. As a control, we analyzed PCR products corresponding to unrearranged substrates from library pools transfected in the absence of *RAG1* and *RAG2* (Figure 6). This control pool shows a bias toward the presence of C nucleotides (the consensus nucleotide at positions 4 and 7–9 of the spacer, and the anticonsensus nucleotide at positions 1 and 6), such that the overall bias of the unselected library is slightly toward the consensus spacer (total consensus/total anticonsensus nucleotides = 1.19), consistent with sequence analysis of untransfected library clones (data not shown). Sequence analysis of amplified SJs reveals an overall enrichment for consensus spacer nucleotides over the unrearranged control (total consensus/total anticonsensus nucleotides = 1.73 for SJs, versus 1.19 for control). Spacer positions 1–5 (adjacent to the heptamer) and 8–11 all show a preference for the consensus nucleotide; the remaining positions show little or no preference for the consensus or in one case (position 7) even an enrichment for the anticonsensus nucleotide (Figure 6, white bars). The strongest preference for consensus is seen at position 5, which shows almost a 3-fold enrichment over the unrearranged control; interestingly, previous mutation analyses have implicated this spacer position as having a role in affecting recombination levels (Fanning et al. 1996; Larijani et al. 1999). In general, the degree of enrichment at any given position reflects the degree to which the consensus nucleotide is represented among the endogenous RSS repertoire (Figure 6) (Ramsden et al. 1994).

To determine whether the preferred spacer sequences for SJ formation and cleavage differ, the library screen was also performed in vitro. To obtain artificial SJs from our biochemical cleavage assays, T4 ligase was added to the deproteinized cleavage products, which circularized the cleavage product containing two signal ends. The sequence analysis of such artificial SJs from two independent cleavage reactions showed that positions 2–5 as well as positions 8–11 of the spacer are enriched for consensus over anticonsensus sequences (Figure 6, black bars). While these observations mirror the SJ formation data, the nucleotide located at position 1 (and to some extent position 3) seems less important for coupled cleavage than for recombination in vivo. Similar to the in vivo experiment, position 5 shows the highest magnitude of enrichment for the consensus (about 4-fold). The differences between the results of the two experimental systems (SJ formation in vivo and cleavage in vitro) could be a reflection of the number of sequences obtained in each type of analysis (200–250) or could represent differences in the nucleotide requirements of spacer participation in cleavage versus SJ formation. Overall, our experiments indicate that spacer effects are largely mediated by the RAG proteins and occur, at least in part, in the first phase of V(D)J recombination: the recognition of the RSSs, their synapsis, and the cleavage step.

### Correlation with a Computational Model for RSS Function

The observation that an RSS spacer can act in concert with the noncritical residues of the heptamer and nonamer to drastically modulate RSS activity suggests the need for models of RSS function that take into account complex functional relationships among the different nucleotides. A predictive algorithm for quantitatively assessing the potential of a given DNA sequence to undergo V(D)J recombination has recently been developed (Cowell et al. 2002, 2003). This algorithm calculates the theoretical recombination potential, or RSS information content (RIC) score, by examining internucleotide relationships within a given DNA sequence.

**Figure 7:**
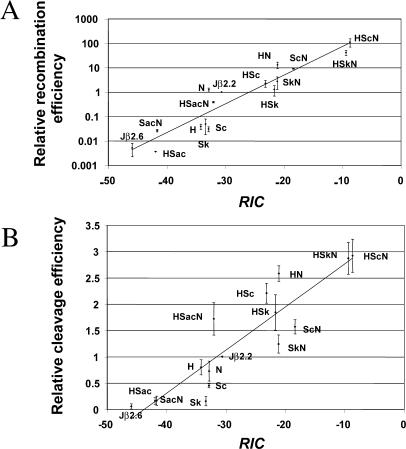
Theoretical Predictions of RSS Qualities The average recombination/cleavage efficiencies obtained in the in vivo experiments (A) and in vitro assays (B) are plotted against the RIC scores for the 12-RSS in the respective recombination substrates. Note that the values obtained from the in vitro cleavage assays were normalized to account for differences in the detection range of individual experiments.

We calculated RIC scores for the hybrid Jβ2.6/consensus RSSs used in this study, and we compared them to the experimental binding, cleavage, and recombination values (Figure 7A and 7B; data not shown). The correlation between RIC scores and our experimental data is striking. The RIC score for Jβ2.6 RSS is below the threshold (−40) for sequences that would be expected to recombine. The addition of consensus heptamer and/or nonamer elements boosts RIC scores, mirroring the increases in binding, cleavage, and SJ formation. Of particular interest is the fact that effects of consensus and anticonsensus spacers on binding/cleavage/recombination are prominently reflected in the RIC scores as well. Intriguingly, RIC scores appear to be more strongly correlated with cleavage (*r_S_* = 0.90) than with binding (*r_S_* = 0.86) and most correlated with SJ formation (*r_S_* = 0.96). The correlations between our experimental data and RIC scores suggest that the failure of Jβ2.6 RSS to recombine and the ability of consensus heptamer, spacer, and nonamer elements to rescue Jβ2.6 RSS activity are functions of how well RSS structure corresponds to that of a preferred sequence. In this case, the selective advantage of the consensus RSS is not limited to a few critical nucleotides in the heptamer or nonamer but, rather, extends throughout the length of the RSS, even in regions (e.g., the spacer) that were previously thought to be unimportant.

Further support for the potential of the RIC score as a theoretical measure for RSS activity arises from our genetic screen. For both the in vivo and the in vitro screens, the mean RIC score of the 12-RSSs in the enriched population is higher than that of the starting pool (data not shown), and those differences are statistically significant (Student's *t* test and the Mann–Whitney test, p<0.0002 for all tests). This indicates that the RIC score is able to predict the quality of RSSs and that this ability is not limited to the well-conserved heptamer and nonamer but also applies to the far more diverse spacer.

## Discussion

RSSs are the DNA elements that direct and control the V(D)J recombination reaction. In the TCR loci, differences in the abilities of individual RSSs to recombine with each other are a significant determinant of variations in the frequencies with which gene elements appear in the mature TCR population (Livàk and Petrie 2002 and references therein). The molecular basis of such differences in intrinsic recombination activities lies in the remarkable sequence diversity of endogenous RSSs. Previous studies using consensus or nearly consensus RSSs suggested that only a handful of absolutely conserved nucleotides in the heptamer and nonamer serve as the major determinants of RSS specificity and function. These studies, however, did not take into account the fact that the vast majority of endogenous RSSs do not contain fully consensus elements; hence, the physiologic roles of lesser-conserved RSS nucleotides are likely of much greater significance than previously estimated.

### Contributions of Individual Elements

Starting from the nonfunctional Jβ2.6 RSS, we asked the following question: what effects do a perfect heptamer, nonamer, or spacer and combinations thereof have in an inactive or poorly active RSS? We show that a number of mutations in noncritical RSS positions are required to convert Jβ2.6 RSS into a highly active 12-RSS or to convert a highly active RSS (H–Sk–N or H–Sc–N) into a completely nonfunctional, pseudogene-type RSS. Our experiments demonstrate that all RSS nucleotides, including the spacer element and the noncritical positions of the heptamer and nonamer, have some sequence-directive roles. In general, we observe that the magnitude of the effects of unfavorable nucleotides in noncritical RSS positions is dependent on the presence of other unfavorable nucleotides. This explains why, in previous studies using largely consensus RSSs, the effects of nonconsensus nucleotides at the noncritical positions were concluded to be less significant (Tonegawa 1983; Hesse et al. 1989).

### Contributions of Individual Nucleotides in Jβ2.6 RSS

The Jβ2.6 RSS heptamer differs from the consensus in the fifth, sixth, and seventh positions; none of these is drastically more important than any other in specifying overall heptamer function (data not shown). The Jβ2.6 RSS nonamer differs from the consensus in the second and fourth positions (see Figure 1), and the G at the fourth position disrupts the poly(A) tract present in the consensus nonamer. Previous footprint analyses and studies on the homologous DNA-binding domain of the bacterial *Hin* recombinase (Feng et al. 1994) suggest that RAG1 may bind the nonamer in the minor groove of this poly(A) tract (Spanopoulou et al. 1996; Akamatsu and Oettinger 1998; Nagawa et al. 1998). Hence, we expected that restoration of the poly(A) tract of the nonamer would have a greater boosting effect on recombination levels than a consensus substitution at the second position. Instead, the opposite is true, regardless of the sequences in the remainder of the RSS (see Figure 3). Having the consensus cytidine at position 2 creates a CA step within the nonamer. Such CA steps have been implicated in alternative DNA structures (Gorin et al. 1995); while previous discussion has focused on the CA steps present at the site of cleavage in the heptamer, it is possible that a single CA step in the nonamer is important for the RAG complex to identify the subsequent downstream poly(A) tract.

### Defects in RAG Binding to Jβ2.6 RSS

Previous binding studies have shown that the nonamer is the key element for initial RAG–RSS interactions and that mutations within the nonamer can strongly reduce or even completely abolish formation of the 12-SC (signal complex) (Hiom and Gellert 1997; Akamatsu and Oettinger 1998). In contrast, mutating the entire heptamer leads only to a partial decrease in 12-SC formation, and, importantly, the absolutely conserved “CAC” triplet contributes only as much to binding as the last four nucleotides of the heptamer (Akamatsu and Oettinger 1998). Our gel-shift studies recapitulate these observations with the Jβ2.6 RSS heptamer and nonamer (see Figure 5). Moreover, a hybrid Jβ2.6/consensus RSS containing a consensus nonamer can promote 12-SC formation as efficiently as the functional Jβ2.2 RSS (see Figure 5). This explains why replacement of the Jβ2.6 RSS nonamer with a consensus nonamer can restore recombination to low but physiologically relevant levels (see Figure 2).

The effect of a consensus spacer on 12-SC formation exhibits striking plasticity (see Figures 2–5). Additionally, in our in vitro screen, the areas of the 12-RSS spacer most highly enriched for consensus nucleotides (see Figure 6) correlate with sites of spacer contacts identified in previous footprinting studies (spacer positions 2–5 and 9–11) (Akamatsu and Oettinger 1998; Nagawa et al. 1998; Swanson and Desiderio 1998; Swanson 2002). Given that the nonamer provides the most important contact surfaces, if strong interactions with the nonamer can form, then the presence of a consensus spacer may allow additional favorable contacts to be established, not only in the spacer itself, but even farther away, in the heptamer. By contrast, an unfavorable spacer (e.g., the Jβ2.6 RSS spacer or Sac) may structurally “insulate” protein–DNA contacts seen in the nonamer, such that potential heptamer contact surfaces that could otherwise contribute to overall 12-SC stability remain hidden. This may explain why a consensus heptamer, in the absence of a good nonamer, is unable to promote formation of a stable 12-SC complex.

Our in vitro cleavage assay integrates the effects of RSS binding, pairing, and actual DNA cleavage. Hence, the differences between the results of binding and cleavage assays suggest that the steps following initial binding (paired complex [PC] formation and DNA cleavage) are also regulated by spacer sequences. PC formation requires the recognition of the partner RSS with respect to its spacer length, and thus it is plausible that the sequence of spacers influences the protein–DNA contacts required for this compatibility test. Since it is within the PC that coordinated, synchronous DNA cleavage takes place (Hiom and Gellert 1998; West and Lieber 1998), it is conceivable that RSSs “communicate” with each other and that their spacer sequences therefore may affect the alignment of the cleavage site with respect to the recombinase active site. Such structural changes may underlie the phenomenon of the “beyond 12/23 rule” that restricts V(D)J recombination of the TCRβ locus, preventing recombination of certain 12–23 RSS pairs and favoring recombination of others (Jung et al. 2003). The 23 bp spacer of the Vβ RSSs is the critical element in dictating the strong preference of Vβ RSSs for the 12-RSS flanking the D segments as compared to the 12-RSS flanking the J segments, and this preference is regulated before or at the cleavage step (Jung et al. 2003). These intriguing findings, however, did not provide experimental insight into how a DNA motif whose sequence had previously been deemed unimportant could paradoxically play such an important role. Our findings provide a framework with which to understand how such an unexpected phenomenon might occur.

Finally, the differences between the in vitro cleavage and in vivo recombination assays indicate an additional role of the spacer sequence in the joining phase of the reaction. This seems plausible, since joining is thought to start with the controlled disassembly of the postcleavage complex in which the four DNA ends, including the RSSs, are held in intimate contact with each other, presumably by the RAG proteins (Hiom and Gellert 1998; Tsai et al. 2002). Spacer sequences might thus be involved in controlling the structure and stability of such complexes.

### Relationship between Spacer Sequence Conservation and Recombination Activity

Based on comprehensive sequence alignments showing a small but significant degree of spacer sequence conservation (Ramsden et al. 1994), a few studies demonstrated reproducible effects of up to 6-fold of naturally occurring spacers on recombination levels (Fanning et al. 1996; Nadel et al. 1998). In transient transfection assays, we infer a much wider range of recombination efficiencies solely due to differences in spacer sequence. Strikingly, we observe that spacer sequence variably affects RSS activity depending on the extent to which each nucleotide of the spacer matches either the most- or the least-conserved nucleotide. This observation resolves some of the apparent discrepancies observed among previously published studies. For example, a poly(G) spacer, which reduces recombination 15-fold compared to a highly active control (Akira et al. 1987), contains one consensus and five anticonsensus residues; by contrast, a spacer containing intermixed G and C residues, which has no effect on recombination activity (Wei and Lieber 1993), contains five consensus and four anticonsensus residues.

### A Structural Basis for the Ability of RAG Proteins to Recombine Highly Diverse RSSs

We find that progressive accumulation of nonconsensus nucleotides within an RSS progressively impairs recombination activity and that, at the less-conserved positions of an RSS, a multitude of nonconsensus nucleotides acting in concert can render the RSS completely inactive. This suggests that the RAG–RSS complex can tolerate or correct for a considerable amount of sequence and/or structural diversity. UV–cross-linking studies previously demonstrated RAG1 and RAG2 cross-linking to the heptamer, particularly near the site of cleavage (Eastman et al. 1999; Mo et al. 1999; Swanson and Desiderio 1999). Footprint analyses of the 12-SC show that complex formation is at least partly blocked by base or phosphate group modification on the spacer side of the heptamer, on both the heptamer- and nonamer-proximal sides of the spacer, and throughout the nonamer (Akamatsu and Oettinger 1998; Nagawa et al. 1998; Swanson and Desiderio 1998; Swanson 2002). The identified contact sites in the spacer coincide with the areas of the spacer that were preferentially found to be consensus type in our genetic screen (see Figure 6). Moreover, the observed recombination efficiencies of our hybrid substrates correlate well with the predicted recombination efficiencies from RIC analyses (see Figure 7A and 7B). Together, these findings support a unifying model in which the RAG proteins establish multiple contacts throughout the length of an RSS (including the spacer) that allow for fine-tuning of activity. Such an extensive network of RAG–RSS contacts within the recombinase complex would create a “structural buffer,” in which unfavorable nucleotides at only a few noncritical positions might be compensated for by favorable protein–DNA interactions at other positions. Conceptually similar models exist for the I-*Ppo*I and I-*Cre*I homing endonucleases, which cleave at recognition sites approximately 20 bp in length (Argast et al. 1998; Jurica et al. 1998), and which can tolerate sequence heterogeneity in cleavage sites. Both I-*Ppo*I and I-*Cre*I form direct sidechain interactions with most of the nucleotides in their recognition sites, and it is believed that the extensive protein–DNA contacts contribute to tolerance of sequence diversity.

Based on our in vivo, in vitro, and in silico analyses, we propose that the RAG–RSS complex contains two distinct types of protein–DNA interactions: “digital” (or binary) interactions of a strictly sequence-specific nature, and “analog” (or multiplicative) contacts that fine-tune the strength of the digital contacts (Travers 1993). Digital interactions are established with those nucleotides for which proper sequence is absolutely critical for activity (e.g., the first three nucleotides of the heptamer and positions 5 and 6 of the nonamer). Analog interactions describe local structural variations brought about by different sequences along the rest of the RSS. Disruption of digital interactions completely precludes complex formation (e.g., a single mutation of a critical residue in the consensus RSS can render it entirely inactive), yet digital interactions alone are not sufficient to establish complex formation (e.g., the critical residues by themselves cannot confer activity to the Jβ2.6 RSS).

This duality in the nature of protein–DNA contacts present within the RAG–RSS recombinase may be applicable to other biological systems, including other transposases, transcription factors, and DNA-binding proteins. In most protein–DNA interaction systems, the target sequence to which a protein binds contains some nucleotides that are absolutely critical, and others that are noncritical. Digital interactions are established with the absolutely conserved nucleotides in the form of sequence-specific binding, conferring a binary specificity; the digital contacts therefore determine whether a protein will bind (+1) or not (0). Analog contacts are then established with the lesser-conserved nucleotides; the analog interactions act as functional multipliers that determine the efficiency of complex stability, yielding a spectrum of binding efficiencies ranging from full activity (1 × A_max_, where A = effect on binding efficiency due to analog interactions) to no activity (0 × A_min_). Hence, the noncritical residues are crucial for determining how well a protein complex can exert its biological function.

By including so many nucleotides as requirements for RSS function, the V(D)J recombination system may have evolved to avoid random cleavage of DNA and translocation errors. If only the critical heptamer and nonamer nucleotides were required for activity, the frequency of cleavage at inappropriate or “cryptic” sites in the genome would be expected to be quite high. By contrast, the required participation of noncritical nucleotides in complex stability safeguards the reaction against uncontrolled cleavage. Hence, from the standpoint of controlled diversification of reaction specificity, it is beneficial for the recombinase to have evolved a spacer with a high degree of sequence heterogeneity, while maintaining intimate contact with the spacer nucleotides via analog interactions. The complex multiplier effect of analog contacts throughout the length of the RSS, superimposed onto specific digital contacts in the heptamer and nonamer, therefore confers upon the recombinase the critical ability to distinguish between inappropriate sites that happen to contain the requisite absolutely conserved nucleotides (e.g., the Jβ2.6 RSS) versus true binding sites whose sequences diverge markedly from the consensus (e.g., most endogenous RSSs).

### Theoretical Predictions of RSS Quality

RIC scores provide a powerful tool for the prediction of RSS quality based on nucleotide sequence. This method generates statistical predictions of RSS function based on the physiologic 12- and 23-RSSs in the mouse antigen receptor gene loci. In our study, RIC scores accurately predicted the relative efficiencies with which RSSs were bound, cleaved, and rearranged (see Figure 7; data not shown). Interestingly, the capacity of RIC models to predict RSS quality is not restricted to sequence variability in the conserved RSS heptamer and nonamer; RIC scores also predict the effects of the RSS spacer sequence on RSS function with considerable accuracy.

It is striking that RIC scores correlate so well with SJ formation, less well with cleavage, and less well still with RSS binding. This supports the idea that individual nucleotides (and groups thereof) make distinct contributions to the different steps of the V(D)J recombination reaction. This concept is consistent with previous findings showing that the nonamer is a major determinant of binding while the influence of the heptamer becomes most apparent at the level of cleavage. Hence, the efficiency with which an RSS recombines represents an integration of its protein–DNA interactions throughout all steps of the reaction, and RIC scores provide a remarkably accurate prediction of this.

RIC models should be useful not only in guiding RSS mutation studies, but also in identifying potential cryptic RSSs in the genome, whose usage could lead to genomic alterations as an initial event leading to chromosomal translocations and cancer (Cowell et al. 2002, 2003). Furthermore, an identical mathematical approach could be useful for predicting binding sites for DNA-binding complexes (e.g., transcription factors) in general, since the algorithm incorporates the combination of both the digital and the analog DNA–protein interactions that determine the biological function of a given protein complex on a potential DNA target.

## Materials and Methods

### 

#### Oligonucleotides and plasmids.

The sequence of oligonucleotides used for cloning of recombination substrates and libraries are presented in Table S1. The oligonucleotides used in the gel-shift experiments are listed in Table S2, and the sequences of oligonucleotides used for PCR (*INNE1*, *CIT4A*, *TL1*, *TL2*, *TL3*, *TL4*, *TL5*, and *TL6*) have been described previously (Eastman et al. 1996; Leu et al. 1997).

The pSJΔ series of substrates for the in vivo recombination and in vitro cleavage assays was created as follows: pSF299 (Fugmann and Schatz 2001) was modified to create p299-Jβ2.6 by replacing the original 12-RSS with a Jβ2.6 12-RSS such that the 12/23-RSS pair is in deletional orientation; for all other substrates, the 12-RSS of p299-Jβ2.6, flanked by *Hind*III and *Sal*I sites, was replaced with the respective annealed oligonucleotides (see Table S1).

To generate the library for the genetic screen, the oligonucleotide *HSCSAC1* was synthesized that contained a 1:1 molar ratio of consensus:anticonsensus nucleotides at each position of the spacer and an additional randomized trinucleotide sequence downstream of the nonamer. The oligonucleotide *SJLIBREV* was annealed, the overhang was filled in using Klenow fragment (New England Biolabs, Beverly, Massachusetts), and the double-stranded fragment was digested with *Hind*III and *Sal*I and ligated into the linearized p299-Jβ2.6 vector. Ligation reactions were transformed into DH5α, colonies were harvested into 120 ml of Luria broth (containing 100 μg/ml ampicillin), and plasmid DNA was prepared after an additional incubation at 37°C at 250 rpm for 15 min.

pEBB, pEBB-RAG1, and pEBB-RAG2 expression constructs have been described elsewhere (Roman et al. 1997).

#### Recombination assays.

Human embryonic kidney 293T cells were transfected with 6 μg of recombination substrate and 3 μg each of pEBB-RAG1 and pEBB-RAG2 using calcium phosphate as described previously (Fugmann and Schatz 2001); for control samples without RAG expression constructs, 6 μg of pEBB was substituted. After 48 h, DNA was recovered by rapid alkaline lysis preparation (RAP) (Hesse et al. 1987). PCR was performed on 10-fold serial dilutions in 20 μl reaction volumes containing 1× Taq buffer (Invitrogen, Carlsbad, California), 2 mM MgCl_2_, 0.1 mM each dNTP, 0.5 μM each oligo, and 0.2 U Taq (Invitrogen). To quantify DNA recovery, the oligonucleotide pair *TL5/TL6* was used for the PCR (94°C for 15 s, 60°C for 15 s, 72°C for 30 s, for 18 cycles). To detect SJs, DNA samples were treated with *Dpn*I, *Mlu*I, and *Xho*I to remove unreplicated and unrecombined plasmids. Oligonucleotides *INNE1* and *CIT4A* were used to amplify SJs (94°C for 15 s, 60°C for 15 s, 72°C for 30 s, for 28 cycles). To detect CJs, RAP samples were treated with *Dpn*I and CJs were amplified using primers *TL2* and *TL3*. All PCR products were electrophoresed on native 4.5% polyacrylamide gels, stained with SYBR green, visualized using a Fluoroimager 595 (Molecular Dynamics, Sunnyvale, California), and quantified using ImageQuant software (Molecular Dynamics).

#### Genetic screen for functional spacer sequences.

293T cells were transfected with the plasmid library and RAG or pEBB constructs as described in the Results. Extrachromosomal DNA was extracted and samples were digested with either *Dpn*I/*Mlu*I/*Xho*I (for cloning of SJs) or *Dpn*I only (for cloning of unrearranged bands in no-RAG controls). PCR was performed using *INNE1* and *CIT4A* primers, and samples were electrophoresed and stained as indicated above. The products corresponding to the appropriate SJ or unrearranged bands were excised, purified, and cloned into pCR2.1 using a TOPO-T/A cloning kit (Invitrogen). DNA was prepared from individual transformed colonies and sequenced.

The in vitro screen was performed using the plasmid library as the substrate in a standard coupled cleavage reaction. After proteinase K digestion, the products were precipitated and dissolved in 100 μl of 1× ligase buffer. T4 DNA ligase (1 μl) (New England Biolabs) was added and the mixture incubated at 16 °C for 4 h to create artificial SJs. The resulting plasmids were treated identically to the plasmids recovered after transfection in the in vivo screen.

#### Protein expression.

Recombinant GST-RAG2, MBP-RAG1, and HMG2 were expressed and purified as described previously (Spanopoulou et al. 1996; Eastman et al. 1999; Rodgers et al. 1999).

#### DNA-binding and cleavage assays.

The body-labeled DNA substrates for the cleavage assay were generated by PCR using the oligonucleotides *TL1*, *TL4*, and the respective recombination substrate as a template. The 12-RSS oligonucleotide substrates used in EMSA were generated by annealing the 5′-end-labeled top strand with an equimolar amount of the unlabeled respective bottom strand (see Table S2). Binding and cleavage reactions were performed as reported previously (Fugmann et al. 2000b), and gels were quantified using a Storm 820 PhosphorImager and ImageQuant software (Molecular Dynamics).

#### RIC score calculation and other computational analysis.

Statistical models of RSS correlation structure have been previously reported (Cowell et al. 2002) (Data S1).

## Supporting Information

Data S1 RIC Score Calculation and Other Computational Analysis (23 KB DOC).

Table S1 Oligonucleotides for Cloning of Recombination Substrates (31 KB DOC).

Table S2 Oligonucleotides for Gel Shift Experiments (23 KB DOC).

## Abbreviations

CJ: coding joint
H: heptamer
Ig: immunoglobulin
N: nonamer
PC: paired complex
PCR: polymerase chain reaction
RAP: rapid alkaline lysis preparation
RIC: recombination signal sequence information content
RSS: recombination signal sequence
Sac: recombination signal sequence spacer
Sc: recombination signal sequence spacer
SC: signal complex
SJ: signal joint
Sk: recombination signal sequence spacer from the VκL8 segment
TCR: T-cell receptor.
