# Supplementary material for: A Functional Analysis of the Spacer of V(D)J Recombination Signal Sequences
Source: PLoS Biol. 2003 Oct 13;1(1):e1. doi: 10.1371/journal.pbio.0000001 (PMC212687; doi:10.1371/journal.pbio.0000001)
Supplement: Data S1 — (23 KB DOC). [file pbio.0000001.sd001.doc]

## RIC score calculation and other computational analysis

Nucleotide positions in RSS not correlated with any other RSS position are included in the models in the form of a marginal probability distribution, *i.e*. the probability of observing nucleotide *X* at RSS position *i, P(X_i_)*. RSS positions that are mutually correlated are included in the models in the form of a joint probability distribution, *e.g*. if the four RSS positions *i*, *j*, *k* and *l* are correlated, their joint probability distribution takes the form *P(X_i_, X_j_, X_k_, X_l_),* the probability of observing, in one RSS, nucleotide *X_i_* at position *i*, *X_j_* at position *j*, *X_k_* at position *k*, and *X_l_* at position *l.* Correlated nucleotides were detected via an iterative statistical procedure discussed in detail in Cowell et al. (2002). For any sequence of appropriate length, the corresponding RSS model (*i.e*. the 12-RSS model for 28-bp sequences and the 23-RSS model for 39-bp sequences) computes the probability that the sequence in question is an RSS, as estimated from the set of physiologic mouse RSS described in (Cowell et al., 2002). Sequences are scored by taking the natural logarithm of this probability; this score is called the RSS information content (*RIC*). *RIC* is computed as follows:

*RIC*_12_ = ln[*P*_1_ *P*_2_ *P*_3,15,25_ *P*_4,5_ *P*_6,28_ *P*_7,8,19_ *P*_9,26_ *P*_10,12_ *P*_11,27_ *P*_13,14,23_ *P*_16,17,18_ *P*_20,21,22_ *P*_24_]

for 12-RS and

*RIC*_23_ = ln[*P*_1_ *P*_2_ *P*_3_ *P*_4,14_ *P*_5,39_ *P*_6_ *P*_7,24,25_ *P*_8,9,21_ *P*_10,16_ *P*_11,12_ *P*_13,22_ *P*_15,23_ *P*_17,18_

*P*_19,27,30,31,32,33,37_ *P*_20,26_ *P*_28,29_ *P*_34,38_ *P*_35,36_]

for 23-RS.

*P*_1_ is the marginal probability distribution for the four nucleotides at position 1, and *P*_3,15,25_ is the joint probability distribution for the 64 triplets at positions 3, 15 and 25. The presence of the joint probability function indicates that these three positions are correlated in the RS alignment.

Cowell, LG, Davila, M, Kepler, TB, and Kelsoe, G (2002) Identification and utilization of arbitrary correlations in models of recombination signal sequences. Genome Biolo*g*y 3: RESEARCH0072.
