# Supplementary material for: A Functional Analysis of the Spacer of V(D)J Recombination Signal Sequences
Source: PLoS Biol. 2003 Oct 13;1(1):e1. doi: 10.1371/journal.pbio.0000001 (PMC212687; doi:10.1371/journal.pbio.0000001)
Supplement: Table S1 — (31 KB DOC). [file pbio.0000001.st001.doc]

**Supporting Table 1.** Oligonucleotides for cloning of recombination substrates

**RSS name nucleotide sequence**

Jβ2.6 JB26R tcgacctcgag gtctg CACAACC CTGTGACTCCCA AGAGAAACC cggcca

JB26 agcttggccg GGTTTCTCT TGGGAGTCACAG GGTTGTG cagac ctcgagg

Jβ2.2 JB22R tcgacctcgag gtttg CACAGTC CTGTGACTCCCA GCACAAACC cggcca

JB22 agcttggccg GGTTTGTGC TGGGAGTCACAG GACTGTG cagac ctcgagg

H JB26C7R tcgacctcgag gtctg CACAGTG CTGTGACTCCCA AGAGAAACC cggcca

JB26C7 agcttggccg GGTTTCTCT TGGGAGTCACAG CACTGTG cagac ctcgagg

Sk JB26GSR tcgacctcgag gtctg CACAACC CTACAGACTGGA AGAGAAACC cggcca

JB26GS agcttggccg GGTTTCTCT TCCAGTCTGTAG GGTTGTG cagac ctcgagg

Sc 26SC2 tcgacctcgag gtctg CACAACC ATACAGCCCTTA AGAGAAACC cggcca

26SC agcttggccg GGTTTCTCT TAAGGGCTGTAT GGTTGTG cagac ctcgagg

N JB26C9R tcgacctcgag gtctg CACAACC CTGTGACTCCCA ACAAAAACC cggcca

JB26C9 agcttggccg GGTTTTTGT TGGGAGTCACAG GGTTGTG cagac ctcgagg

HN JB26C7C9R tcgacctcgag gtctg CACAGTG CTGTGACTCCCA ACAAAAACC cggcca

JB26C7C9 agcttggccg GGTTTTTGT TGGGAGTCACAG CACTGTG cagac ctcgagg

HSk JB26C7GSR tcgacctcgag gtctg CACAGTG CTACAGACTGGA AGAGAAACC cggcca

JB26C7GS agcttggccg GGTTTCTCT TCCAGTCTGTAG CACTGTG cagac ctcgagg

HSc 26HSC2 tcgacctcgag gtctg CACAGTG ATACAGCCCTTA AGAGAAACC cggcca

26HSC agcttggccg GGTTTCTCT TAAGGGCTGTAT CACTGTG cagac ctcgagg

SkN JB26GSC9R tcgacctcgag gtctg CACAACC CTACAGACTGGA ACAAAAACC cggcca

JB26GSC9 agcttggccg GGTTTTTGT TCCAGTCTGTAG GGTTGTG cagac ctcgagg

ScN 26SCN2 tcgacctcgag gtctg CACAACC ATACAGCCCTTA ACAAAAACC cggcca

26SCN agcttggccg GGTTTTTGT TAAGGGCTGTAT GGTTGTG cagac ctcgagg

HSkN JB26GOODR tcgacctcgag gtctg CACAGTG CTACAGACTGGA ACAAAAACC cggcca

JB26GOOD agcttggccg GGTTTTTGT TCCAGTCTGTAG CACTGTG cagac ctcgagg

HScN 26HSCN2 tcgacctcgag gtctg CACAGTG ATACAGCCCTTA ACAAAAACC cggcca

26HSCN agcttggccg GGTTTTTGT TAAGGGCTGTAT CACTGTG cagac ctcgagg

Sac RSAC2 tcgacctcgag gtctg CACAACC CAGATCTAGGAG AGAGAAACC cggcca

RSAC1 agcttggccg GGTTTCTCT CTCCTAGATCTG GGTTGTG cagac ctcgagg

HSac RHSAC2 tcgacctcgag gtctg CACAGTG CAGATCTAGGAG AGAGAAACC cggcca

RHSAC1 agcttggccg GGTTTCTCT CTCCTAGATCTG CACTGTG cagac ctcgagg

SacN RSACN2 tcgacctcgag gtctg CACAACC CAGATCTAGGAG ACAAAAACC cggcca

RSACN agcttggccg GGTTTTTGT CTCCTAGATCTG GGTTGTG cagac ctcgagg

HSacN RHSACN2 tcgacctcgag gtctg CACAGTG CAGATCTAGGAG ACAAAAACC cggcca

RHSACN agcttggccg GGTTTTTGT CTCCTAGATCTG CACTGTG cagac ctcgagg

**Supplemental Table 1 (continued).** Oligonucleotides for cloning of recombination substrates

**RSS name nucleotide sequence**

N(2) 26N2C2 tcgacctcgag gtctg CACAACC CTGTGACTCCCA ACAGAAACC cggcca

26N2C1 agcttggccg GGTTTCTGT TGGGAGTCACAG GGTTGTG cagac ctcgagg

N(4) 26N4C2 tcgacctcgag gtctg CACAACC CTGTGACTCCCA AGAAAAACC cggcca

26N4C1 agcttggccg GGTTTTTCT TGGGAGTCACAG GGTTGTG cagac ctcgagg

ScN(2) 26SCN22 tcgacctcgag gtctg CACAACC ATACAGCCCTTA ACAGAAACC cggcca

26SCN21 agcttggccg GGTTTCTGT TAAGGGCTGTAT GGTTGTG cagac ctcgagg

ScN(4) 26SCN42 tcgacctcgag gtctg CACAACC ATACAGCCCTTA AGAAAAACC cggcca

26SCN41 agcttggccg GGTTTTTCT TAAGGGCTGTAT GGTTGTG cagac ctcgagg

SkN(2) 26SN2C2 tcgacctcgag gtctg CACAACC CTACAGACTGGA ACAGAAACC cggcca

26SN2C1 agcttggccg GGTTTCTGT TCCAGTCTGTAG GGTTGTG cagac ctcgagg

SkN(4) 26SN4C2 tcgacctcgag gtctg CACAACC CTACAGACTGGA AGAAAAACC cggcca

26SN4C1 agcttggccg GGTTTTTCT TCCAGTCTGTAG GGTTGTG cagac ctcgagg

HN(2) 26HN22 tcgacctcgag gtctg CACAGTG CTGTGACTCCCA ACAGAAACC cggcca

26HN21 agcttggccg GGTTTCTGT TGGGAGTCACAG CACTGTG cagac ctcgagg

HN(4) 26HN42 tcgacctcgag gtctg CACAGTG CTGTGACTCCCA AGAAAAACC cggcca

26HN41 agcttggccg GGTTTTTCT TGGGAGTCACAG CACTGTG cagac ctcgagg

HScN(2) 26HSCN22 tcgacctcgag gtctg CACAGTG ATACAGCCCTTA ACAGAAACC cggcca

26HSCN21 agcttggccg GGTTTCTGT TAAGGGCTGTAT CACTGTG cagac ctcgagg

HScN(4) 26HSCN42 tcgacctcgag gtctg CACAGTG ATACAGCCCTTA AGAAAAACC cggcca

26HSCN41 agcttggccg GGTTTTTCT TAAGGGCTGTAT CACTGTG cagac ctcgagg

H(5)N JB26H5N2 tcgacctcgag gtctg CACAGCC CTGTGACTCCCA ACAAAAACC cggcca

JB26H5N1 agcttggccg GGTTTTTGT TGGGAGTCACAG GGCTGTG cagac ctcgagg

H(5)Sc 26H5SC2 tcgacctcgag gtctg CACAGCC ATACAGCCCTTA AGAGAAACC cggcca

26H5SC agcttggccg GGTTTCTCT TAAGGGCTGTAT GGCTGTG cagac ctcgagg

H(6)Sc JB26H6SC2 tcgacctcgag gtctg CACAATC ATACAGCCCTTA AGAGAAACC cggcca

JB26H6SC1 agcttggccg GGTTTCTCT TAAGGGCTGTAT GATTGTG cagac ctcgagg

H(7)Sc JB26H7SC2 tcgacctcgag gtctg CACAACG ATACAGCCCTTA AGAGAAACC cggcca

JB26H7SC1 agcttggccg GGTTTCTCT TAAGGGCTGTAT CGTTGTG cagac ctcgagg

H(5)ScN 26H5SCN2 tcgacctcgag gtctg CACAGCC ATACAGCCCTTA ACAAAAACC cggcca

26H5SCN1 agcttggccg GGTTTTTGT TAAGGGCTGTAT GGCTGTG cagac ctcgagg

library SJLIBREV cgtcactcgagacgcgt gtctg

HSCSAC1 gcaggaagcttnnnggccg GGTTTCTCT YWMSKRSWKYWK CACTGTG cagac

acgcgtctcgagtgacg
