# Supplementary material for: A Functional Analysis of the Spacer of V(D)J Recombination Signal Sequences
Source: PLoS Biol. 2003 Oct 13;1(1):e1. doi: 10.1371/journal.pbio.0000001 (PMC212687; doi:10.1371/journal.pbio.0000001)
Supplement: Table S2 — (23 KB DOC). [file pbio.0000001.st002.doc]

**Supporting Table 2.** Oligonucleotides for gel shift experiments

**RSS name nucleotide sequence**

Jβ2.6 JBETA26T gatctggcctg gtctg CACAACC CTGTGACTCCCA AGAGAAACC tgcactcgagcggag

JBETA26B ctccgctcgagtgca GGTTTCTCT TGGGAGTCACAG GGTTGTG cagac caggccagatc

H HT gatctggcctg gtctg CACAGTG CTGTGACTCCCA AGAGAAACC tgcactcgagcggag

HB ctccgctcgagtgca GGTTTCTCT TGGGAGTCACAG CACTGTG cagac caggccagatc

N NT gatctggcctg gtctg CACAACC CTGTGACTCCCA ACAAAAACC tgcactcgagcggag

NB ctccgctcgagtgca GGTTTTTGT TGGGAGTCACAG GGTTGTG cagac caggccagatc

Sc SCT gatctggcctg gtctg CACAACC ATACAGCCCTTA AGAGAAACC tgcactcgagcggag

SCB ctccgctcgagtgca GGTTTCTCT TAAGGGCTGTAT GGTTGTG cagac caggccagatc

HN HNT gatctggcctg gtctg CACAGTG CTGTGACTCCCA ACAAAAACC tgcactcgagcggag

HNB ctccgctcgagtgca GGTTTTTGT TGGGAGTCACAG CACTGTG cagac caggccagatc

HSc HSCT gatctggcctg gtctg CACAGTG ATACAGCCCTTA AGAGAAACC tgcactcgagcggag

HSCB ctccgctcgagtgca GGTTTCTCT TAAGGGCTGTAT CACTGTG cagac caggccagatc

ScN SCNT gatctggcctg gtctg CACAACC ATACAGCCCTTA ACAAAAACC tgcactcgagcggag

SCNB ctccgctcgagtgca GGTTTTTGT TAAGGGCTGTAT GGTTGTG cagac caggccagatc

HScN HSCNT gatctggcctg gtctg CACAGTG ATACAGCCCTTA ACAAAAACC tgcactcgagcggag

HSCNB ctccgctcgagtgca GGTTTTTGT TAAGGGCTGTAT CACTGTG cagac caggccagatc

Jβ2.2 JBETA22T gatctggcctg gtttg CACAGTC CTGGAAATGCTG GCACAAACC tgcactcgagcggag

JBETA22B ctccgctcgagtgca GGTTTGTGC CAGCATTTCCAG GACTGTG caaac caggccagatc

HSac HSACT gatctggcctg gtctg CACAGTG CAGATCTAGGAG AGAGAAACC tgcactcgagcggag

HSACB ctccgctcgagtgca GGTTTCTCT CTCCTAGATCTG CACTGTG cagac caggccagatc

SacN SACNT gatctggcctg gtctg CACAACC CAGATCTAGGAG ACAAAAACC tgcactcgagcggag

SACNB ctccgctcgagtgca GGTTTTTGT CTCCTAGATCTG GGTTGTG cagac caggccagatc

HSacN HSACNT gatctggcctg gtctg CACAGTG CAGATCTAGGAG ACAAAAACC tgcactcgagcggag

HSACNB ctccgctcgagtgca GGTTTTTGT CTCCTAGATCTG CACTGTG cagac caggccagatc
